# Optimization of enzymatic esterification of dihydrocaffeic acid with hexanol in ionic liquid using response surface methodology

**DOI:** 10.1186/s13065-017-0276-2

**Published:** 2017-05-26

**Authors:** Somayeh Gholivand, Ola Lasekan, Chin Ping Tan, Faridah Abas, Leong Sze Wei

**Affiliations:** 1 Department of Food Technology, University Putra Malaysia, 43400 Serdang, Selangor Malaysia; 2 Department of Food Science, University Putra Malaysia, 43400 Serdang, Selangor Malaysia; 3 Laboratory of Natural Products, Institute of Bioscience, University Putra Malaysia, 43400 Serdang, Selangor Malaysia

**Keywords:** Enzymatic esterification, Hexyl dihydrocaffeate, Candida antartica lipase (Novozyme 435), Ionic liquid, Response surface methodology (RSM)

## Abstract

**Background:**

Developing an efficient lipophilization reaction system for phenolic derivatives could enhance their applications in food processing. Low solubility of phenolic acids reduces the efficiency of phenolic derivatives in most benign enzyme solvents. The conversion of phenolic acids through esterification alters their solubility and enhances their use as food antioxidant additives as well as their application in cosmetics.

**Results:**

This study has shown that lipase-catalyzed esterification of dihydrocaffeic acid with hexanol in ionic liquid (1-butyl-3-methylimidazoliumbis (trifluoromethylsulfonyl) imide) was the best approach for esterification reaction. In order to achieve the maximum yield, the process was optimized by response surface methodology (RSM) based on a five-level and four independent variables such as: dosage of enzyme; hexanol/dihydrocaffeic acid mole ratio; temperature and reaction time. The optimum esterification condition (Y = 84.4%) was predicted to be obtained at temperature of 39.4 °C, time of 77.5 h dosage of enzyme at 41.6% and hexanol/dihydrocaffeic acid mole ratio of 2.1.

**Conclusion:**

Finally, this study has produced an efficient enzymatic esterification method for the preparation of hexyl dihydrocaffeate in vitro using a lipase in an ionic liquid system. Concentration of hexanol was the most significant (p < 0.05) independent variable that influenced the yield of hexyl dihydrocaffeate.

**Graphical abstract:**
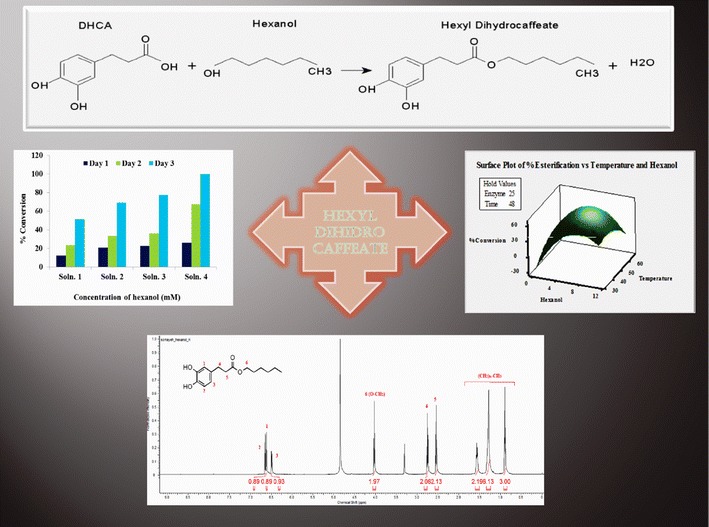
Synthesis of different Hexyl dihydrocaffeates in ionic liquid

## Background

Due to the useful biological properties of phenolic acids, they have been reported to have widespread applications in the pharmaceutical, cosmetics and food industries [1–5]. Phenolic acids are compounds which act as hydrogen donors, singlet oxygen quenchers and reducing agents [6]; therefore, they are classified as antioxidants. Anti-atherosclerotic, anti-cancer or anti-carcinogenic, anti-mutagenic, anti-viral, anti-bacterial and anti-inflammatory activities are the other important properties of many phenolic compounds [7]. Most of the phenolic acids are hydrophilic compounds; thus, they exhibit low solubility and stability in oil-based formulae which has restricted their application in various industries [2]. However, to apply naturally occurring phenolic acids in oil-based formulation and food processing, the esterification strategy of their carboxylic acid group with suitable aliphatic alcohol is necessary.

The conversion of phenolic acids through esterification alters their solubility and enhances their use as food antioxidant additives as well as their application in cosmetics. phenolic acids trans-esterification with chemical method reduces their product yield since they are heat sensitive and susceptible to oxidation under certain pH [8]. Therefore, lipase-catalyzed reaction in organic solvent media under mild conditions can be used as an alternative synthetic method. In spite of improving the conversion yield by this method, the existence of a few technical difficulties during the enzyme-catalyzed esterification of phenolic acids has been noticed such as their low solubility in the presence of some organic solvent media (e.g. in hexane) where the enzyme is active or reversed behavior in other solvents (e.g. DMSO). Hence, to overcome the above problems, the other suitable synthetic pathway is needed. Ionic liquids have been introduced as a green synthesis media, instead of organic solvents, for the enzymatic transformation of various substances [9].

Ionic liquids (ILs) which are made from bulky cation and a small anion, have been classified as tunable designer solvents with very low volatility and high thermal stability [10]. These solvents have attracted the attention of organic researchers as greener replacements to conventional organic media in order to facilitate sustainable chemistry. The low toxicity of some of the ionic liquids [11, 12], reusability, ecofriendly nature of ionic liquids and also their ability to improve the enzyme stability and selectivity are the other advantages of ILs for the bio-catalytic modification of phenolic compounds [13–17]. Several groups have also reported the use of ionic liquids for the enzymatic transformation of flavonoid [9], glucose [18], phenolic glycosides and other compounds [19–22].

**Fig. 1:**
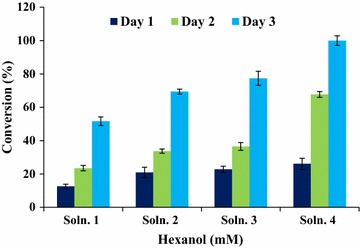
Esterification of DHCA in 1–3 days by varying molar ratio of dihydrocaffeic acid to hexanol: solution 1 (1:2), solution 2 (1:4), solution 3 (1:8) and solution 4 (1:16) keeping all other parameters constant [at temperature 55 °C and 250 rpm in ionic liquid (1-butyl-3 methylimidazolium)bis (trifluoromethylsulfonyl) imide]

In the present study, the enzymatic esterification of dihydrocaffeic acid (DHCA) with 1- hexanol (Fig. 1) as a model reaction was carried out in ionic liquid [1-butyl-3-methylimidazoliumbis (trifluoromethylsulfonyl) imide]. DHCA is a degradation product of caffeic acid with potent antioxidant properties [2]. In fact, it is formed by human intestinal bacteria as hydrogenated analogue metabolite of caffeic acid and eriocitrin [23]. To assist the enzymatic esterification reaction between DHCA and hexanol, response surface methodology (RSM) was used to investigate the reaction processes and elucidate the relationships between the optimized factors (substrate concentration; dosage of the enzyme, reaction time and temperature) on the degree of esterification as a response variable. RSM is a valuable tool used to determine the optimum levels of two or more treatment variables. In addition; RSM is a useful approach for reducing the experimental runs, time and cost in comparison with one factor at a time for hexyl dihydrocaffeate production. It has important applications in the design, development and formulation of new products, as well as in the improvement of existing product design [24].

## Results and discussion

### Effect of hexanol concentration on the conversion yield of hexyl dihydrocaffeate

Results of the esterification of DHCA during a 3 days period are shown in Fig. 1. Increasing the hexanol concentration resulted in higher conversion of DHCA to its hexyl ester. Nearly 100% conversion yield was obtained when the DHCA to hexanol mole ratio of 1:16 was used for 3 days. Although using high dosage of alcohol was particularly useful for obtaining a higher conversion yield for the antioxidant (hexyl dihydrocaffeate) but its separation and purification from the residual unreacted alcohol in solutions containing the DHCA/hexanol mole ratio greater than 1:2 posed serious problems. Therefore, the synthesis of the hexyl dihydrocaffeate was carried out in solutions containing lower DHCA/alcohol ratio (i.e. less than 1:2 ratio) in order to overcome the above drawback. This range of acid/alcohol mole ratio (less than 1:2) was used for optimization of esterification.

To perform the reaction at high temperatures, Novozyme 435 was selected as one of the thermo stable immobilized enzymes since it has optimum reaction temperature of 40–60 °C and a longer half time [25]. Higher temperature not only decreases the system viscosity which increases the mass transfer [26] but also improves the kinetics of enzymatic reaction which resulted in higher conversion yield. Selection of the suitable range for all factors affecting the conversion of DHCA was based on either the preliminary experiments or literature data [27].

### Fitting the response surface model

Multivariate regression analysis was performed using response surface analysis to fit the mathematical model to the experimental values aiming at high composite desirability and optimal region for the response variable studied, to describe the relationship between four factors and response variable and finally determine the optimum conditions for the synthesis of the natural antioxidant in ionic liquid system. The response surface analysis permitted the development of an empirical relationship where response (Y) was measured as a function of substrate mole ratio (X_1_), enzyme amount (X_2_), reaction time (X_3_), reaction temperature (X_4_) and also predicted as the sum of constant (β_0_), four first-order effects (linear terms in X_1_, X_2_, X_3_ and X_4_), four second-order effects (quadratic terms in ${\text{X}}_{1}^{2} ,{\text{X}}_{2}^{2} ,{\text{X}}_{3}^{2} \;{\text{and}}\;{\text{X}}_{4}^{2}$) and four interaction effects (interactive terms in X_1_X_2_, X_1_X_3_, X_1_X_4_ and X_2_X_4_). The results of the analysis of variance revealed a “goodness of fit”. In the reduced model, only statistical significant (p < 0.05) terms were included.

The fitted quadratic regression (1), illustrating the degree of conversion of DHCA to its ester as a function of four factors, was determined as follows:

1 $$ \begin{aligned} {\mathbf{Y}} &= - 300.110 + 18.654\,{\mathbf{X}}_{{\mathbf{1}}} + 3.831\,{\mathbf{X}}_{{\mathbf{2}}} + 1.182\,{\mathbf{X}}_{{\mathbf{3}}} + 8.425\,{\mathbf{X}}_{{\mathbf{4}}}  \\ &\quad - \, 1.409\,{\mathbf{X}}_{{\mathbf{1}}}^{{\mathbf{2}}} - 0.052\,{\mathbf{X}}_{{\mathbf{2}}}^{{\mathbf{2}}} - 0.011\,{\mathbf{X}}_{{\mathbf{3}}}^{{\mathbf{2}}} - 0.065\,{\mathbf{X}}_{{\mathbf{4}}}^{{\mathbf{2}}} + 0.249\,{\mathbf{X}}_{{\mathbf{1}}} {\mathbf{X}}_{{\mathbf{2}}}  \\ & \quad + 0.058\,{\mathbf{X}}_{{\mathbf{1}}} {\mathbf{X}}_{{\mathbf{3}}} - 0.182\,{\mathbf{X}}_{{\mathbf{1}}} {\mathbf{X}}_{{\mathbf{4}}} - 0.038\,{\mathbf{X}}_{{\mathbf{2}}} {\mathbf{X}}_{{\mathbf{4}}} \end{aligned} $$

According to the results given in Eq. 1, the model obtained for the response variable described the linear, quadratic and interaction effects of four independent variables affecting the yield. The predicted regression coefficient of adjusted model along with its lack of fit test, R^2^ adjusted and R^2^ value is presented in Table 1. The final reduced model which has been fitted based on F test at the 5% confidence level of the regression model (p < 0.05) resulted in a second-order polynomial model for the response variable studied. It should be noted that the full quadratic equation was fitted for predicting the degree of esterification value.

**Table 1:** ANOVA and regression coefficient of the first and second degree polynomial regression model

Sl. no.	Terms	P value^a^	F ratio	Regression coefficient	Response
1	Constant		–	β_0_	−300.110
2	X_1_	0.000	29.06	β_1_	18.654
3	X_2_	0.007	9.84	β_2_	3.831
4	X_3_	0.002	14.65	β_3_	1.182
5	X_4_	0.000	22.91	β_4_	8.425
6	${\text{X}}_{1}^{2}$	0.000	83.20	β_12_	−1.409
7	${\text{X}}_{2}^{2}$	0.007	9.64	β_22_	−0.052
8	${\text{X}}_{3}^{2}$	0.002	14.56	β_32_	−0.011
9	${\text{X}}_{4}^{2}$	0.001	15.01	β_42_	−0.065
10	X_1_X_2_	0.000	24.52	β_12_	0.249
11	X_1_X_3_	0.015	7.63	β_13_	0.058
12	X_1_X_4_	0.003	13.12	β_14_	−0.182
13	X_2_X_4_	0.036	5.34	β_24_	−0.038
R^2^					0.95
R^2^ (adj)					0.91
(p value)					0.000^a^
(F value)					51.26

Concentration of hexanol (×10^−4^ mM) X_1_, enzyme amount (%) X_2_, reaction time (h) X_3_ and reaction temperature X_4_

^a^Significant (p < 0.05)

The unity of the R^2^ value means that the model is strong and also has a better prediction of the response [28]. On the other, the coefficient of determination (R^2^) provided the quality and satisfactory fit to the second-order polynomial model. It has been reported that for the model to have goodness of fit (predicted response variables), the R^2^ should be greater than 0.80 [29]. In the current study, the high values of R^2^ adjusted (0.91) and R^2^ (0.95) indicated the high variability in the response variable studied and high significance in model respectively, and as a result, these guaranteed an adequate fitting of the polynomial model to the experimental data. Also, the lack of fit (*F* value), which measures the fitness of models, resulted in no significant *F* value (p > 0.05) in terms of the response variables studied.

In addition, Table 1 shows that the linear terms of the four independent variables had significant positive effects on conversion while at the same time indicating a negative effect on the quadratic terms. Interaction effects between two factors namely: hexanol-enzyme and hexanol-time were positively related to the percent of conversion value. Conversely, response value was negatively influenced by interaction between hexanol-temperature and enzyme-temperature. These significant terms should be considered as the primary independent variables for evaluating the variation of conversion yield.

In accordance with Table 1, the most significant independent factor affecting the variation of conversion yield was found in the quadratic term for concentration of hexanol. Furthermore, the probability (p) of the regression model (0.000) confirmed that the model was highly significant for the response variable. Also, Table 1 clearly revealed that the esterification of dihydrocaffeic acid as a response variable was significantly (*p* < 0.05) influenced by the linear and quadratic terms of all studied factors, followed by the interaction effects of hexanol-enzyme, hexanol-time, hexanol-temperature and enzyme-temperature variables.

### Optimization procedure

Graphical and numerical optimization procedures were employed to predict the precise level of four factors resulting in the desirable goal of response variable. An optimal treatment was shown by response surface plotting of the values. Using 3D response surface of the regression model for the graphical interpretation of the factor interactions has been highly recommended [30–32].

**Fig. 2:**
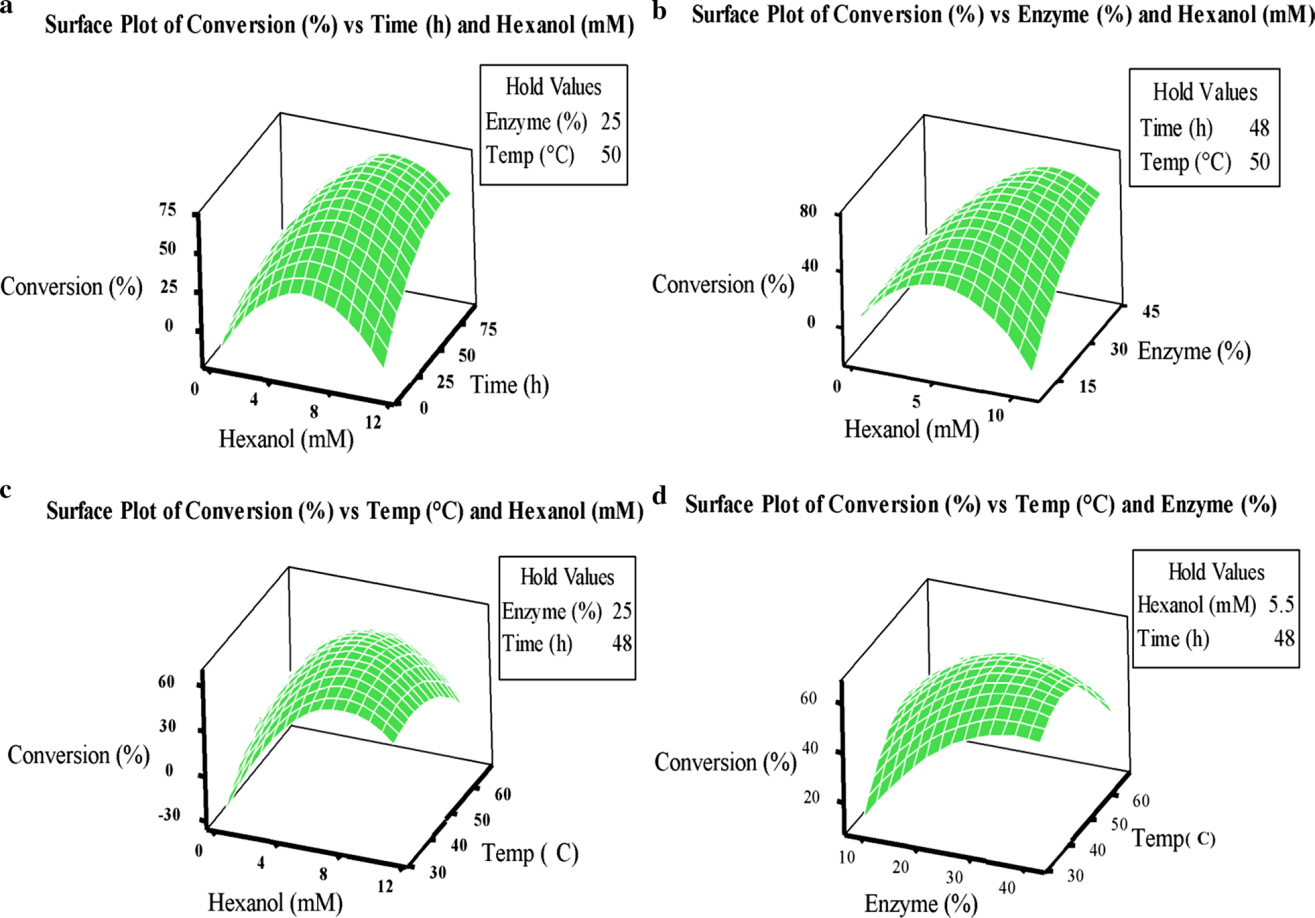
Three-dimensional response surface plots (**a**–**d**) of the conversion of dihydrocaffeic acid to hexyl dihydrocaffeate

Figure 2 indicated the three-dimensional (3D) response surface for two factors at a constant value of the other two factors. The 3D diagrams can be used to better understand the significant (*p* < 0.05) interaction effect between two independent variables and response as well as locate their optimum levels. As it is evident from the surface plots (a, b, c and d), by increasing the concentration of hexanol, the response variable (hexyl dihydrocaffeate) increased. Further increase in hexanol concentration (>6 mM) significantly decreased the percent conversion of hexyl dihydrocaffeic acid (HDHCA). Also the conversion yield was increased with increased enzyme amount, time and temperature up to 40 °C.

**Fig. 3:**
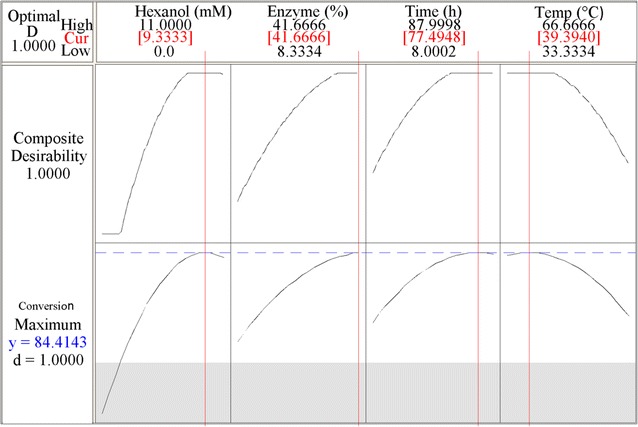
Numerical response optimizer

The response optimizer was employed to generate the optimal set of four factors leading to the desired criteria of response variable. The conversion of dihydrocaffeic acid would be viewed as an optimal product if the goal employed for numerical or graphical optimization led to a maximum degree of esterification. The optimum esterification conditions were predicted to be obtained at temperature of 39.4 °C, time of 77.5 h dosage of enzyme at 41.6% and concentration of hexanol at 9.3 × 10^−4^ mM using response optimizer and response surface plots. As it can be seen in Fig. 3, the composite and the individual desirability for the degree of esterification of phenolic compound (dihydrocaffeic acid) were found to be one.

### Model verification

Comparison was made between the experimental data of the response and the fitted values predicted by the response regression model (Table 2) using two-sample T test for checking the adequacy of the response surface equation. The results exhibited no significant (*p* > 0.05) difference between the predicted data and actual values. Proximity of the observed values and predicted data and also the high correlation coefficient (0.95) affirmed that the corresponding response surface model was adequate in predicting variations of response variable as a function of esterification conditions. However, the close results of the predicted and experimental values under the optimum area revealed the appropriateness of the model.

**Table 2:** Central composite design: factor (X_i_), response variable (Y) and residual

Run	Concentration of hexanol^a^ (mM × 10^−4^)	Enzyme amount^b^ (%)	Reaction time (h)	Reaction temperature (°C)	Conversion (%)	Predicted (%)	Residual
	X_1_	X_2_	X_3_	X_4_	Y	Y_0_	Y − Y_0_
12^c^	5.5 (0)	25.0 (0)	48.0 (0)	50.0 (0)	65.131	62.222	2.909
11	8.8 (+1)	35.0 (+1)	24.0 (−1)	60.0 (+1)	25.054	28.459	−3.404
4	2.2 (−1)	15.0 (−1)	24.0 (−1)	60.0 (+1)	17.151	23.714	−6.563
23	2.2 (−1)	15.0 (−1)	72.0 (+1)	40.0 (−1)	12.597	16.561	−3.964
5	2.2 (−1)	35.0 (+1)	24.0 (−1)	40.0 (−1)	18.375	9.751	8.623
26	8.8 (+1)	15.0 (−1)	72.0 (+1)	60.0 (+1)	29.213	36.068	−6.855
3	8.8 (+1)	35.0 (+1)	72.0 (+1)	40.0 (−1)	81.140	78.966	2.174
14^c^	5.5 (0)	25.0 (0)	48.0 (0)	50.0 (0)	64.554	62.222	2.332
1	2.2 (−1)	35.0 (+1)	72.0 (+1)	60.0 (+1)	19.431	24.580	−5.149
19	8.8 (+1)	15.0 (−1)	24.0 (−1)	40.0 (−1)	21.519	11.621	9.897
6	5.5 (0)	25.0 (0)	8.0 (−1.66)	50.0 (0)	17.720	25.369	−7.649
25	11.0 (1.66)	25.0 (0)	48.0 (0)	50.0 (0)	35.044	34.562	0.482
13^c^	5.5 (0)	25.0 (0)	48.0 (0)	50.0 (0)	62.571	60.415	2.156
28^c^	5 (0)	25.0 (0)	48.0 (0)	50.0 (0)	60.401	60.415	−0.014
22	5.5 (0)	8.3 (−1.66)	48.0 (0)	50.0 (0)	35.341	34.698	0.643
7	5.5 (0)	25.0 (0)	87.9 (1.66)	50.0 (0)	66.911	59.798	7.113
20	5.5 (0)	41.6 (1.66)	48.0 (0)	50.0 (0)	55.932	57.111	−1.179
15	5.5 (0)	25.0 (0)	48.0 (0)	66.6 (1.66)	45.865	41.460	4.405
27	0.0 (−1.66)	25.0 (0)	48.0 (0)	50.0 (0)	0.000	1.018	−1.018
9	5.5 (0)	25.0 (0)	48.0 (0)	33.3 (−1.66)	38.220	43.160	−4.94
30	2.2 (−1)	35.0 (+1)	24.0 (−1)	60.0 (+1)	21.574	20.005	1.569
2	8.8 (+1)	15.0 (−1)	72.0 (+1)	40.0 (−1)	42.416	48.361	−5.945
18	8.8 (+1)	35.0 (+1)	72.0 (+1)	60.0 (+1)	69.420	65.198	4.222
10	8.8 (+1)	15.0 (−1)	24.0 (−1)	60.0 (+1)	15.065	13.174	1.891
29^c^	5.5 (0)	25.0 (0)	48.0 (0)	50.0 (0)	66.363	69.144	−2.781
16	2.2 (−1)	35.0 (+1)	72.0 (+1)	40.0 (−1)	24.885	28.172	−3.287
8	2.2 (−1)	15.0 (−1)	24.0 (−1)	40.0 (−1)	15.505	11.986	3.519
24	2.2 (−1)	15.0 (−1)	72.0 (+1)	60.0 (+1)	49.382	42.135	7.247
17	8.8 (+1)	35.0 (+1)	24.0 (−1)	40.0 (−1)	53.586	56.072	−2.486
21^c^	5.5 (0)	25.0 (0)	48.0 (0)	50.0 (0)	65.196	69.144	−3.948

^a^Substrate molar ratio was selected correspondingly to 0.5 − 2(2.2–8.8 × 10^−4^ mM) hexanol to dihydrocaffeic acid (4.4 × 10^−4^ mM)

^b^15–35% relative to the total weight of substrates

^c^ Center point

### Characterization of the purified product

#### FTIR analysis for hexyl dihydrocaffeate

The results of the hexyl dihydrocaffeate FTIR with spectrum in the 4000–280 cm^−1^ regions are presented in Table 3. The bands at 3385–3502 cm^−1^ could be attributable to the stretching of the hydroxyl functional group of catechol moiety. The adsorption bands at about 2900 cm^−1^ with two branches can be attributed to the C=H stretching of aromatic and aliphatic, while the peak at 1704 cm^−1^ was assigned to C=O stretching of the ester. The bands found at wavelengths lower than 1608 cm^−1^ revealed the presence of aromatic ring in the structure of the resultant ester. Also, the bands between 1261 and 1008 are related to C–O stretching of the ester (Table 3).

**Table 3:** Representative FTIR spectra (cm^−1^) of dihydrocaffeic acid ester

Frequency range (cm^−1^)	Frequency (cm^−1^)			Functional groups
	DHCA^a^		Hexyl dihydrocaffeate	
3500–3200	3338		3502^b^, 3386	O–H stretching
3000–2850	2925–2869		2917–2856	C–H stretching
1760–1690	1668		1705	C=O stretching
1600–1400	1525–1441		1608–1534	C=C stretching
1320–1000	1207–1286		1192–1261	C–O stretching
700 ± 20	–	–	728	Long chain (C–H)

^a^Dihydrocaffeic acid

^b^H-bond lattice

#### NMR analysis for hexyl dihydrocaffeate and other alkyl esters

**Fig. 4:**
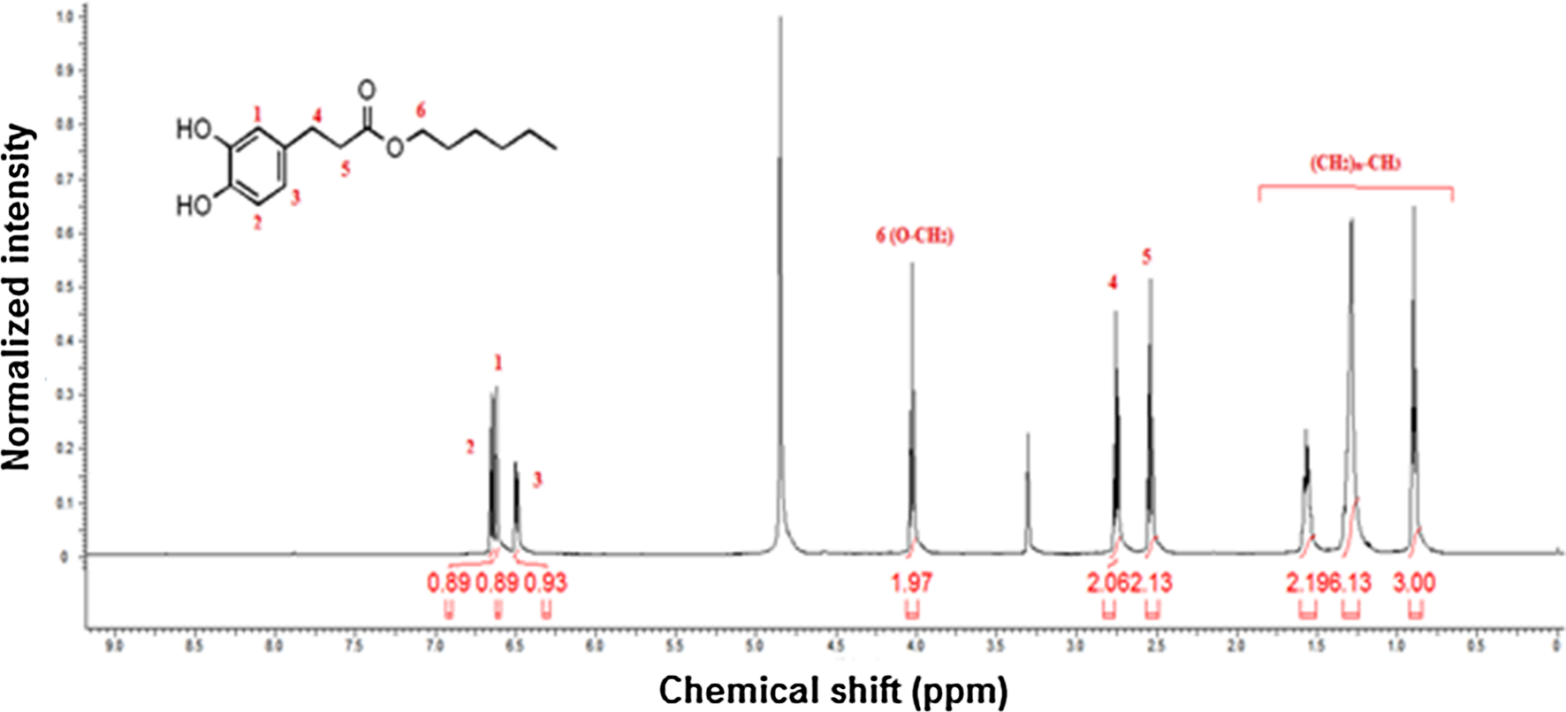
NMR spectrum of hexyl dihydrocaffeate

The synthesized antioxidant ester (hexyl dihydrocaffeate) was identified by 1H NMR (500 MHz, (METHANOL-d4)) and its result is presented in Fig. 4. Chemical shifts (δ) were given in ppm while coupling constants were in Hz. The result of the ester is: ^1^H NMR δ ppm: 0.90 (t, J = 6.99 Hz, 3 H), 1.20–1.36 (m, 6 H), 1.50–1.62 (m, 2 H), 2.54 (t, J = 7.57 Hz, 2 H), 2.75 (t, J = 7.60 Hz, 2 H), 4.03 (t, J = 6.70 Hz, 2 H), 6.49 (dd, J = 8.15, 1.75 Hz, 1 H), 6.62 (d, J = 1.75 Hz, 1 H), 6.65 (d, J = 8.15 Hz, 1 H).

## Conclusion

The central composite design (CCD) was shown to be a valuable tool for optimizing the esterification conditions of dihydrocaffeic acid. Concentration of hexanol was the most significant (p < 0.05) independent variable affecting the conversion yield. Results have shown that hexyl dihydrocaffeate yield was significantly (p < 0.05) affected by the linear and quadratic terms of all the factors studied. Moreover, the optimization procedure showed that the optimum region with the highest desirability (D = 1) was achieved at temperature of 39.4 °C, time of 77.5 h dosage of enzyme at 41.6% and concentration of hexanol at 9.3 × 10^−4^ mM.

## Experimental

### Materials

Ionic liquids consisting: 1-butyl-3-methylimidazoliumbis (trifluoromethylsulfonyl) imide with purity of 98% was acquired from Sigma-Aldrich (Milan, Italy). Immobilized lipase (triacylglycerol hydrolase, EC 3.1.1.3; Novozyme 435 from C.*antarctica* supported on acrylic resin beads was obtained from Novo Nordisk Bio-industrials, Inc. (Bagsvaerd, Denmark). 3,4 Dihydroxycaffeic acid (DHCA), hexanol, and methanol with the highest available purity purchased from Sigma-Aldrich (Milan, Italy). 3 Å molecular sieves (10–20 Mesh beads) were obtained from Fluka (Fluka, Neu-Ulm, Germany). Chloroform, diethyl ether and all solvents were of analytical and HPLC grades purchased from Fisher Scientific, Inc. (Slangerup, Denmark). Thin-layer chromatography (TLC) with a silica gel 60F254 plate no. 5715 was obtained from Merck (Darmstadt Germany).

### Experimental design

The effect of four factors [i.e. X_1_ (dihydrocaffeic acid to hexanol molar ratio of 1:0.5–1:2); X_2_ (immobilized-enzyme amount 15–35% by the total weight of substrates); X_3_ (reaction time of 24–72 h) and X_4_ (reaction temperature of 40–60 °C)] were selected for the synthesis of hexyl dihydrocaffeate as a response variable and was assessed using RSM (Table 4). Central composite design (CCD), as a statistical method, was employed to investigate the major and combined effects of all independent variables on the degree of esterification, to generate a model between variables and finally, to evaluate the effect of these parameters in order to optimize the reaction conditions for the synthesis of hexyl dihydrocaffeate enzymatically leading to the desired goal. MINITAB v16 statistical package (Minitab Inc., State College, PA, USA) software was applied for generating experimental design matrix, data analysis and optimization procedure.

**Table 4:** Levels of independent variables established according to central composite design for enzymatic esterification of hexyl dihydrocaffeate condition level (coded and, un coded)

Independent variable	Independent variable level				
	Low (−1)	Medium (0)	High (+1)	Axial (−1.66)	Axial (+1.66)
Reaction time^a^	24	48	72	8.0	88.0
Reaction Temp^b^	40	50	60	33.3	66.7
Conc. of hexanol^c^	2.2	5.5	8.8	0.0	11.0
Enzyme amount^d^	15	25	35	8.3	41.6

^a^Reaction time (h)

^b^Reaction temperature (°C)

^c^Concentration of hexanol (substrate molar ratio was selected correspondingly to 0.5 − 2((2.2–8.8) × 10^−4^ mM) of hexanol to dihydrocaffeic acid (4.4 × 10^−4^ mM)

^d^15–35% relative to the total weight of substrates

As presented in Table 2, 30 experimental runs along with center point which was repeated six times, were assigned using a second order composite design taking into account four independent variables at five levels of each factor [30]. The treatments were randomized in order to reduce the impact of unexplained variability in the actual response owing to extraneous parameters.

### Statistical analysis

Analysis of variance and regression analysis were carried out to determine statistically significant model terms and regression coefficients, fitting of mathematical models to the experimental data aiming at an overall optimal region and high composite desirability for the response variable. Multiple regression coefficients were employed by applying the least-squares technology [33] to predict the linear, quadratic polynomial models and also interactive effects between tested parameters for response function. The mathematical relationship among factors and response was expressed in the quadratic polynomial Eq. 2:

2 $$ Y = \beta_{0} + \mathop \sum \limits_{i = 1}^{4} \beta_{i} \chi_{i} + \mathop \sum \limits_{i = 1}^{4} \beta_{ii} \chi_{i}^{2} + \mathop \sum \limits_{i = 1}^{3} \mathop \sum \limits_{j = i + 1}^{4} \beta ij xixj $$

where: Y is the predicted response value, *β*_*0*_ is constant, *βi*, *βii* and *βij* are the linear, quadratic and interaction regression coefficients of RSM model, and Xi and Xj are the factor variables.

The individual linear, quadratic and interaction effects and also regression coefficients terms of tested parameters were presented by the ANOVA results. According to the F-ratio at P-value of 0.05; the significance of the equation parameters for response based on model analysis, coefficient of determination (R^2^ = at least 0.80) analysis as outlined and the suitability of the model were evaluated [29, 34, 35].

### Optimization procedure

The optimization procedure was employed to acquire the optimal levels of four independent variables (X_1_, X_2_, X_3_ and X_4_) after producing the polynomial regression equation concerning the factors studied. By choosing desired goal for the variable, numerical optimization was performed to evaluate the exact optimum level of factors leading to the desired enzymatic synthesis conditions in terms of percent of dihydrocaffeic acid conversion. Optimum conditions that depended on the factors, were achieved through the predicted equation evaluated using RSM. Moreover, for deducing workable optimal conditions, a graphical technique was applied [36, 37]. Generally, the three-dimensional (3D) response surface plots were employed for visualizing the relationship between experimental levels for each independent variable and the response and also for deducing the optimal conditions [30, 38]. In order to interpret graphical interaction of the four factors studied (graphical optimization), the 3D response surface plots were plotted by altering two variables in the experimental range and keeping the other two variables constant at the center point.

### Model verification

Model validation was done theoretically by comparing the experimental data and predicted values. This was employed to assess the adequacy of the final reduced model equation obtained using two-sample T test. There must be close agreement between the predicted and experimental values and no significant difference existing for model adequacy validation. Experiments under the suggested optimum conditions were tested to verify the suitability of response model for predicting optimum data as well.

### Enzymatic esterification of dihydrocaffeic acid with hexanol in ionic liquid

The enzymatic esterification of DHCA was performed in ionic liquid (1-buthyl-3-methylimidazoliumbis (trifluoromethylsulfonyl) imide). A fixed amount of DHCA (4.4 × 10^−4^ mM) with different quantities of hexanol, according to the substrate mole ratios presented in Table 2, were introduced into the test tubes which contained various dosages of the powdered *C. antarctica* lipase in ionic liquid as a medium. Typical reactions were conducted in the presence of activated 3Å molecular sieves as by-product (water) eliminating substance. The reactions were performed in an incubator equipped with a shaker operating at 250 rpm, under various temperatures and times (Table 2). With the removal of the biocatalyst, the reaction was terminated and then, quantitative analysis of the synthesized esters was carried out by HPLC.

### Ester purification

The alkyl dihydrocaffeate esters were obtained by extracting each reacted solution in diethyl ether. The ether layer was dried over sodium sulfate and filtered [39]. In each case, the filtrate was evaporated to dryness under reduced pressure. The extract was applied onto a silica gel column (2.5 × 30 cm, fractions of 50 mL), which washed with 200 mL of chloroform (100%) and 400 mL of (99:1–98:2%) chloroform–methanol mixture respectively for eluting the unreacted substrates. The formed ester product was detected by thin-layer chromatography (TLC) [40] or HPLC methods.

### Degree of esterification

The following equation was employed [28] to calculate the degree of esterification from the HPLC profile:

$$ {\text{Degree}}\;{\text{of}}\;{\text{esterification }}\left( \% \right) = \varvec{A}_{\text{tpe}} /\left( { 1: 4 6 2\,\varvec{A}_{\text{tp}} + \varvec{A}_{\text{tpe}} } \right) $$

where *A*_tp_ is the total peak area of dihydrocaffeic acid, *A*_tpe_ is the total peak area of hexyl dihydrocaffeate and 1.462 is the ratio of the average molecular weight of hexyl dihydrocaffeate to the average molecular weight of dihydrocaffeic acid.

### Characterization of purified product

#### HPLC analysis

The reaction components were diluted with methanol and filtered using syringe filter (0.22 µm). A 10 µL aliquot was taken from the reaction mixture and injected in HPLC (Agilent 1200 series, Waldbrunn, Germany) equipped with a C18 reverse-phase capillary column (250 × 4.6 mm, 5 µL) as well as an ultraviolet detector at wavelength range of 200–325 which was applied for DHCA evaluation. The injected samples were eluted by mobile phases including: 90% of solvent A and 10% of solvent B [namely methanol (pre-dried over 3 Å molecular sieves) and 0.75% of acetic acid in distilled water respectively] at a flow rate of 1 mL/min for 16 min [41].

#### Fourier transforms infrared (FTIR) and nuclear magnetic resonance (NMR) analysis

Fourier transform infrared (FTIR) and nuclear magnetic resonance (NMR) spectroscopy were used for characterization of each alkyl dihydrocaffeate. The functional groups of each synthesized compound were identified by Perkin Elmer spectrum 100 Series spectrometer (United Kingdom) facilitated with a mid-infrared detector-DTGS (deuterated triglycine sulphate). Each synthesized product was dispersed in potassium bromide pellet and compressed into a disc by pressure. Then the IR spectrum of each sample was recorded. The resultant FTIR spectrum in the 4000–280 cm^−1^ regions with resolution of 4 cm^−1^. Nuclear magnetic resonance (NMR) spectra were recorded on Varian 500 MHz-NMR Spectrometer. The synthesized product was dissolved in methanol-d4 and the chemical shifts were given on the parts per million scales with TMS as an internal standard.

## Abbreviations

ANOVA: analysis of variance
CCD: central composite design
D: desirability
DHCA: dihydrocaffeic acid
DMSO: dimethyl sulfoxide
DTGS: deuterated triglycine sulphate
ILs: ionic liquids
NMR: nuclear magnetic resonance
FTIR: Fourier transforms infra-red
